# Microbial production of novel sulphated alkaloids for drug discovery

**DOI:** 10.1038/s41598-018-26306-7

**Published:** 2018-05-22

**Authors:** Eitaro Matsumura, Akira Nakagawa, Yusuke Tomabechi, Shinichi Ikushiro, Toshiyuki Sakaki, Takane Katayama, Kenji Yamamoto, Hidehiko Kumagai, Fumihiko Sato, Hiromichi Minami

**Affiliations:** 1 Research Institute for Bioresources and Biotechnology, Ishikawa Prefectural University, 1-308 Suematsu, Nonoichi, Ishikawa, 921–8836 Japan; 2 Department of Applied Chemistry, School of Engineering, Tokai University, 4-1-1 Kitakaname, Hiratsuka, Kanagawa 259–1292 Japan; 3 Department of Biotechnology, Faculty of Engineering, Toyama Prefectural University, 5180 Kurokawa, Imizu, Toyama, 939–0398 Japan; 4 Division of Integrated Life Science, Graduate School of Biostudies, Kyoto University, Sakyo-ku, Kyoto, 606–8502 Japan

## Abstract

Natural products from plants are useful as lead compounds in drug discovery. Plant benzylisoquinoline alkaloids (BIAs) exhibit various pharmaceutical activities. Although unidentified BIAs are expected to be of medicinal value, sufficient quantities of such BIAs, for biological assays, are sometimes difficult to obtain due to their low content in natural sources. Here, we showed that high productivity of BIAs in engineered *Escherichia coli* could be exploited for drug discovery. First, we improved upon the previous microbial production system producing (*S*)-reticuline, an important BIA intermediate, to obtain yields of around 160 mg/L, which was 4-fold higher than those of the previously reported highest production system. Subsequently, we synthesised non-natural BIAs (*O*-sulphated (*S*)-reticulines) by introducing human sulphotransferases into the improved (*S*)-reticuline production system. Analysis of human primary cells treated with these BIAs demonstrated that they affected a biomarker expression in a manner different from that by the parent compound (*S*)-reticuline, suggesting that simple side-chain modification altered the characteristic traits of BIA. These results indicated that highly productive microbial systems might facilitate the production of scarce or novel BIAs and enable subsequent evaluation of their biological activities. The system developed here could be applied to other rare natural products and might contribute to the drug-discovery process as a next-generation strategy.

## Introduction

Many medicinal compounds have been discovered from natural resources, with some in wide use as popular medications. Currently, ~60% of approved small molecular medicines are related to natural products: 64 natural products, 299 natural-product derivatives, and 268 natural-product mimics^1^. The active components of these pharmaceuticals accumulate in sufficient amounts in nature to enable scientific evaluation; therefore, it is relatively easy to harvest the source and assess the possibilities for medical use. By contrast, certain natural compounds anticipated as novel drug candidates occur in low concentrations in nature, making identification of the useful activities of these rare natural products difficult. Therefore, the low content of some target compounds makes drug discovery from natural products difficult^1^. Advances in biotechnology and synthetic biology allow microbial production of difficult-to-obtain compounds, including plant secondary metabolites^2^. The antimalarial drug artemisinin isolated from plants was produced by a microbial system and used for medical treatment to contribute to the stabilization of price and supply^3^. Engineered microbes can produce appreciable amounts of scarce natural compounds, thereby enabling the synthesis of the derived novel and synthetic compounds, as well as the validation of their activities.

Benzylisoquinoline alkaloids (BIAs) constitute a family of major secondary metabolites of plants, of which many members, such as morphine and codeine, exhibit strong pharmaceutical effects. Similar to other natural compounds, unknown and scarce BIAs are expected to be a source for drug discovery; BIAs with novel functions have been demonstrated as promising anticancer drug candidates, including novel bisbenzylisoquinoline alkaloids used as ingredients for crude drugs^4,5^. Because most BIAs are synthesised via (*S*)-reticuline^6^, various BIAs can be produced if enough (*S*)-reticuline is available; however, (*S*)-reticuline is an intermediate that can be easily converted to other BIAs in plants and does not accumulate in sufficient levels. Additionally, it is difficult to purify (*S*)-reticuline from natural resources, such as the latex of opium poppies, without contamination of other BIAs possessing similar chemical properties. Furthermore, inexpensive synthesis of (*S*)-reticuline by chemical methods is also difficult because of the requirements for troublesome regiospecific and chiral-specific reactions.

Microbial production of (*S*)-reticuline from simple sugars has been accomplished in *E. coli*^7^ and yeast systems^8,9^, which have been developed for the total biosynthesis of opioids, like thebaine and hydrocodone^10,11^. *E. coli* is one of the most popular bacteria used for producing chemicals such as amino acids and organic acids, because of the ease of manipulation, availability of genetic tools, and knowledge of its physiology. In this study, we improved the yields of the current *E*. *coli*-production system of (*S*)-reticuline by 4-fold^7^. Furthermore, we successfully produced two kinds of synthetic BIAs, 7-*O*- and 3′-*O*-sulphated (*S*)-reticulines, by introducing human sulphotransferases (hSULTs) into the improved bacterial platform. Finally, we assessed the biological activity of these BIAs by treating human primary cells and measuring the levels of biomarkers associated with various disease models^12,13^.

## Results

### An improved microbial platform for efficient (*S*)-reticuline production

**Figure 1:**
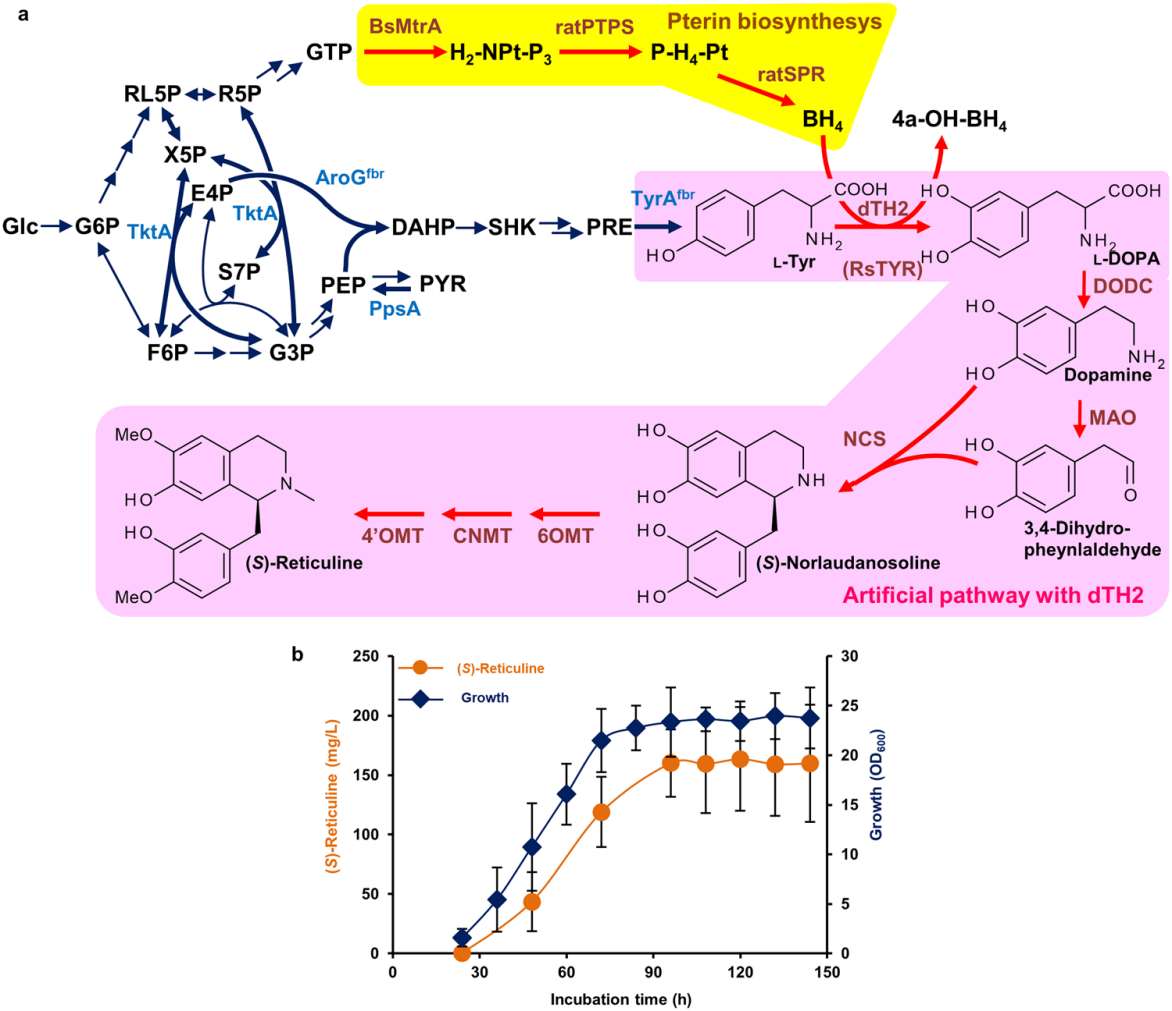
Alternative platform for benzylisoquinoline alkaloid (BIA) biosynthesis in *Escherichia coli*. (**a**) Biosynthetic platform for BIAs in *E. coli*. Bold arrows indicate the modified reactions. The colour codes are as follows: blue, inherent enzymes; red, heterologous enzymes. The pink- and yellow-shaded zones indicate the artificial pathway for BIA synthesis and for *de novo* BH_4_ synthesis, respectively. Abbreviations of the compounds and enzymes are as follows: G6P, d-glucose 6-phosphate; F6P, d-fructose 6-phosphate; RL5P, d-ribulose 5-phosphate; R5P, d-ribose 5-phosphate; X5P, d-xylose 5-phosphate; E4P, d-erythrose 4-phosphate; S7P, d-sedoheptulose 7-phosphate; G3P, d-glyceraldehyde 3-phosphate; PEP, phosphoenolpyruvate; PYR, pyruvate; DAHP, 3-deoxy-d-arabino-heptulosonate-7-phosphate; SHK, shikimate; PRE, prephenate; H_2_-NPt-P_3_, 7,8-dihydroneopterin triphosphate; P-H_4_-Pt, 6-pyruvoyltetrahydropterin; BH_4_, tetrahydrobiopterin; 4a-H-BH_4_, 4a-hydroxytetrahydrobiopterin; TyrA^fbr^, chorismate mutase-prephenate dehydrogenase (feedback-resistant); AroG^fbr^, 2-dehydro-3-deoxyphosphoheptonate aldolase; TktA, transketolase; PpsA, phosphoenolpyruvate synthetase; dTH2, tyrosine hydroxylase; RsTYR, tyrosinase; DODC, l-DOPA decarboxylase; MAO, monoamine oxidase; 6OMT, norcoclaurine 6-*O*-methyltransferase; 4′OMT, 3′-hydroxy-*N*-methyl-(*S*)-coclaurine 4′-*O*-methyltransferase; CNMT, coclaurine *N*-methyltransferase; BsMtrA, GTP cyclohydrolases I; ratPTPS, 6-pyruvoyltetrahydropterin synthase; and ratSPR, sepiapterin reductase. (**b**) Fermentative production of (*S*)-reticuline with strain EM353 in a jar fermenter. (*S*)-Reticuline was obtained from seven independent experiments; in three experiments, production was analysed at 24–96 h and in four experiments at 72–144 h. Error bars represent SD.

The previous *E. coli* platform used tyrosinase for l-Tyr hydroxylation to l-3,4-dihydroxyphenylalanine (l-DOPA) (Fig. 1a); however, tyrosinase shows degradative activity against intermediates, such as l-DOPA, dopamine, and norlaudanosoline^14^, resulting in reduced (*S*)-reticuline productivity. In the yeast system used for BIA production, tyrosine hydroxylase (TH) was successfully used for l-DOPA synthesis via the tetrahydrobiopterin (BH_4_)-synthetic pathway^9^. To avoid intermediate degradation, we established an alternative *E. coli* platform using TH from *Drosophila melanogaster* (dTH2), which is similar to the yeast system. dTH2 requires BH_4_ as a cofactor, which *E. coli* cannot produce^15^ (Supplementary Fig. S1a). For functional expression of dTH2 in *E. coli*, a *de novo* biosynthetic pathway for BH_4_ production was constructed using three codon-optimised genes encoding guanosine triphosphate (GTP) cyclohydrolase I (MtrA), 6-pyruvoyltetrahydropterin synthase (PTPS), and sepiapterin reductase (SPR) as previously reported^16^ (Supplementary Fig. S2 and Supplementary Table S1). To analyse the contributions of the three genes to BH_4_ synthesis, *E*. *coli* transformants were constructed with plasmids containing the complete gene set and another lacking a single BH_4_-biosynthesis gene (Supplementary Fig. S1b). The strain harbouring the complete set of the BH_4_-biosynthesis genes produced biopterins (126 ± 14 μM), whereas the single-gene-lacking strains produced little or no biopterins (Supplementary Fig. S1c), indicating that the full set of BH_4_-biosynthesis genes was required for effective l-DOPA production. The highest l-DOPA productivity was observed in the culture of dTH2 with the complete set of the BH_4_-biosynthesis genes (Supplementary Fig. S1d); therefore, we decided to use all three enzymes for BH_4_ synthesis.

To produce (*S*)-reticuline, we constructed the engineered *E*. *coli* strain EM353, which possessed a tyrosine-overproducing pathway, a pathway producing dopamine from l-tyrosine along with the BH_4_-synthesis pathway, and a pathway producing (*S*)-reticuline from dopamine. (*S*)-reticuline production in this strain required 14 genes, which were cloned separately into four vectors (Supplementary Fig. S3 and Supplementary Table S1). EM353 was cultured in a jar fermenter with 30 g/L glucose. (*S*)-reticuline production started at 24 h after inoculation and the yields reached a plateau beyond 96 h (Fig. 1b). The maximum yield of (*S*)-reticuline was 163.5 ± 43.6 mg/L at 120 h after inoculation. This titre was four-folds higher than that of a previously reported system using tyrosinase (40 mg/L from glucose)^7^, indicating that dTH2 was more suitable for BIA production in *E. coli*.

### Production of unnatural BIAs using microbial (*S*)-reticuline

Semi-synthetic modification of small natural products is often effective for increasing or altering pharmaceutical activity, with many approved medicines having been derived from natural products with simple modifications^1^. Sulphate conjugation is a feasible modification capable of extending medical effects, because some medicines, such as the anti-hypertensive agent triamterene and the hair-growth stimulant minoxidil, are activated by sulphate conjugation^17^. Additionally, the position of sulphate conjugation in dopamine confers different effects: dopamine 4-*O*-sulphate has vasopressor activity, whereas dopamine 3-*O*-sulphate has depressor activity^17^. Therefore, sulphate-conjugated plant secondary metabolites, such as BIAs, might also exhibit extended or alternative activities. Although naturally sulphated secondary metabolites have been studied, sulphate conjugation of BIAs has not been applied for drug discovery of novel drug candidates.

**Figure 2:**
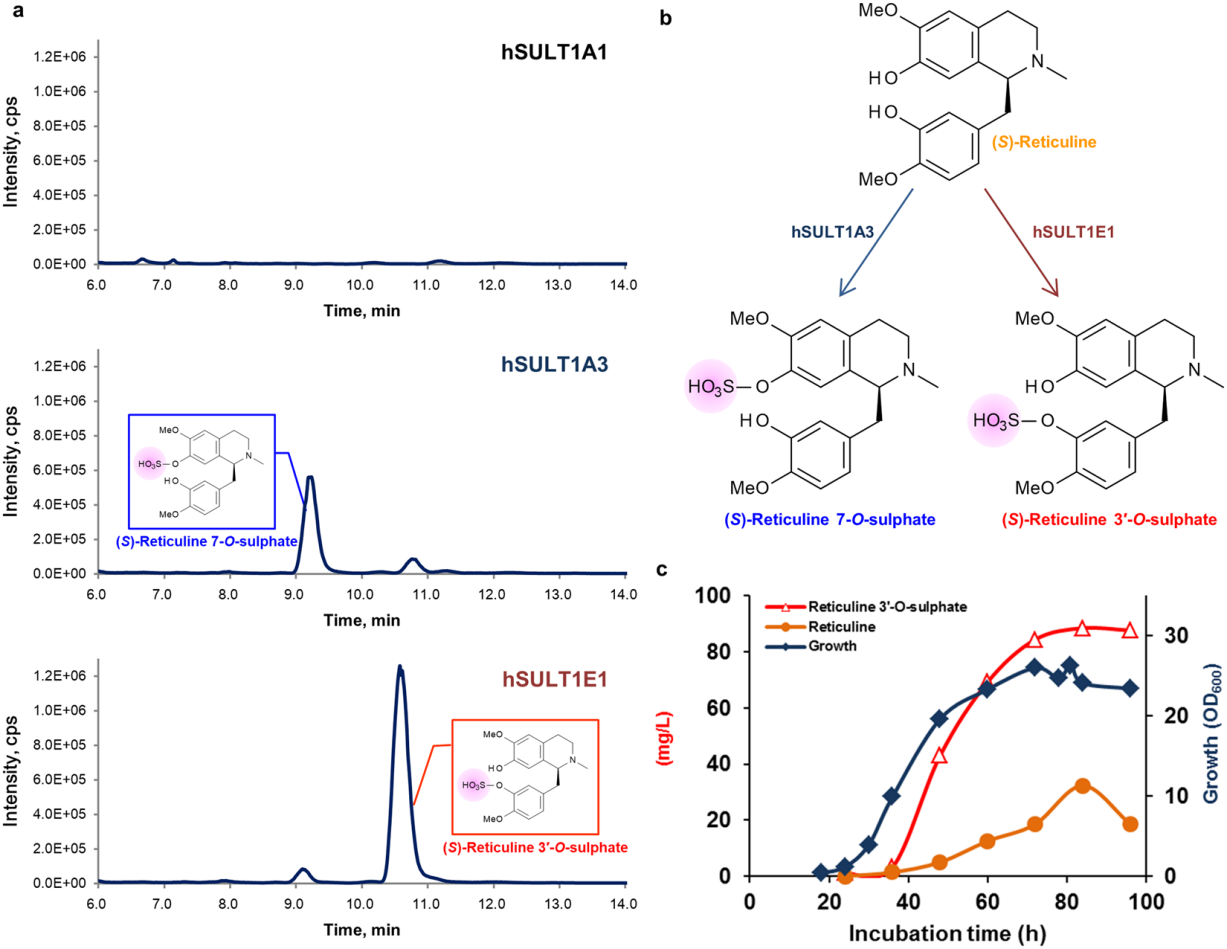
Production of (*S*)-reticuline *O*-sulphates. (**a**) LC-MS analyses of the products of *hSULT*-expressing *Escherichia coli* with (*S*)-reticuline. Sulphated *O*-(*S*)-reticulines were analysed by mass spectrometry in negative ionization mode (*m*/*z* = 408). (**b**) Scheme of (*S*)-reticuline *O*-sulphate production with hSULTs. (**c**) Fermentative production of (*S*)-reticuline 3′-*O*-sulphate in a jar fermenter.

In chemical synthesis, selective modification of a compound with multiple hydroxyl groups is complicated, because it requires protection/deprotection steps. By contrast, enzymatic reactions generally have high regiospecificity. Mammalian SULTs exhibit relatively broad substrate specificity and were expected to convert BIAs to sulphated forms. To evaluate mammalian SULT sulphation activity for (*S*)-reticuline in *E. coli*, we constructed strains expressing three kinds of hSULT genes (*hSULT1A1*, *hSULT1A3*, and *hSULT1E1*), and these strains were cultured in (*S*)-reticuline-containing medium (Fig. 2a and Supplementary Table S2). Liquid chromatography tandem mass spectrometry (LC-MS/MS) analysis of the culture medium showed that (*S*)-reticuline monosulphates were produced in the culture of *hSULT1A3op*- and *hSULT1E1op*-expressing strains; however, in either culture, we could not detect (*S*)-reticuline disulphate, which was also expected to be produced by hSULTs. Although both 3′-*O*- and 7-*O*-sulphated (*S*)-reticuline were found in the cultures of *hSULT1A3op*- and *hSULT1E1op*-expressing strains, hydroxyl-group selectivity was quite different (Fig. 2a). hSULT1A3 could convert (*S*)-reticuline to mainly (*S*)-reticuline 7-*O*-sulphate, and hSULT1E1 dominantly produced (*S*)-reticuline 3′-*O*-sulphate (Fig. 2a and b and Supplementary Fig. S4). The signal intensities of these compounds in MS analysis were stronger than parental compound (*S*)-reticuline, indicating that addition of a sulphate group enhances negative ionization (Supplementary Fig. S5). High resolution mass analysis confirmed that these compounds were sulphated (*S*)-reticulines (Supplementary Figure S6). As expected, hSULTs were successfully used for (*S*)-reticuline sulphation, and we proceeded to produce sulphate-conjugated (*S*)-reticuline using *hSULT1A3op* and *hSULT1E1op*.

### Fermentative production of (*S*)-reticuline *O*-sulphates in *E. coli*

To produce (*S*)-reticuline *O*-sulphates in a one-pot culture, *hSULT1E1op* and *hSULT1A3op* genes were introduced into the (*S*)-reticuline-fermentative strain EM353, resulting in EM437 and EM459, respectively. EM437 produced (*S*)-reticuline 3′-*O*-sulphate from glucose in a one-pot culture at titres reaching 90.9 mg/L (Fig. 2c) and displaying a productivity of 44.7% (*S*)-reticuline in a large-scale culture. We found 32.3 mg/L (19.7% of the yields by EM353) of unreacted (*S*)-reticuline, indicating that *hSULT1E1op* in EM437 did not have enough activity for complete conversion to (*S*)-reticuline 3′-*O*-sulphate. The remaining (*S*)-reticuline (35.6%) would be degraded and/or not produced in EM437. Although a one-pot culture of the *hSULT1A3op-*co-expressing strain EM459 did not produce (*S*)-reticuline 7-*O*-sulphate from glucose, (*S*)-reticuline 7-*O*-sulphate was obtained at 55.8 mg/L (Supplementary Table S3) when the *hSULT1A3op*-expressing strain EM407 was cultured in medium containing (*S*)-reticuline purified from the EM353 culture. Therefore, efficient production of regiospecifically sulphated (*S*)-reticulines was accomplished by improving the (*S*)-reticuline productivity of the pre-existing method, which did not require any expensive substrates, rather, only a simple sugar. The *E. coli*-based BIA platform showed great potential for the practical fermentation of BIAs, including novel BIAs not previously identified in nature.

### Biological activities of (*S*)-reticuline *O*-sulphates in human cells

**Figure 3:**
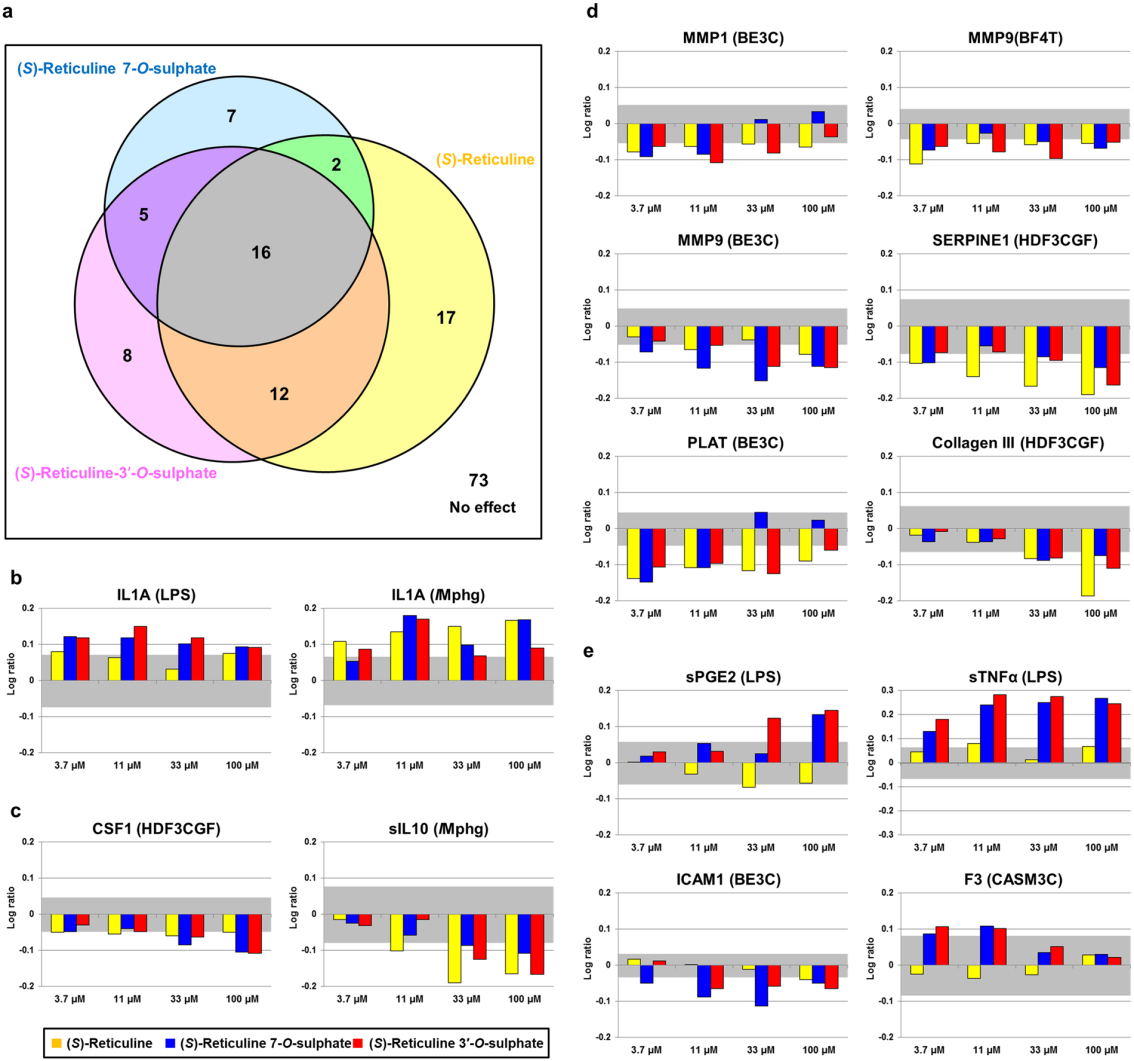
Effect of (S)-reticuline sulphation on biomarker expression in a human primary cell-based assay. (**a**) Venn diagram of overlapping bioactivities of (*S*)-reticuline and (*S*)-reticuline *O*-sulphates on 140 biomarkers from the BioMAP system (Supplementary Table S4). (**b**–**d**) Examples of biomarkers affected by all three BIAs with the same tendency, including inflammation-related (**b**), immunomodulatory (**c**), and tissue-remodelling-related (**d**) biomarkers. (**e**) Examples of significant biomarkers changed in a sulphation-specific manner. The parentheses indicate the BioMAP system used for respective biomarker readouts: LPS, cardiovascular disease; /Mphg, cardiovascular disease; HDF3CGF, fibrosis; BE3C, lung disease; BF4T, asthma and allergy; and CASM3C, restenosis model systems. Abbreviations of the biomarkers are as follows: IL1A, iterleukin-1α; sTNF-α, soluble tumour necrosis factor-α; CSF1, macrophage colony-stimulating factor; sIL10, soluble iterleukin-10; MMP1, matrix metalloproteinase-1; MMP9, matrix metalloproteinase-9; PAI-I, plasminogen-activator inhibitor-1; PLAT, tissue plasminogen activator; sPGE2, soluble prostaglandin E_2_; and ICAM1, intercellular-adhesion molecule-1. The Y-axis shows the log_10_ expression ratios of the readout relative to the dimethyl sulfoxide control. The grey zone indicates the threshold of each biomarker.

The high productivity of novel compounds prompted us to evaluate their biological activities. (*S*)-reticuline and the non-natural BIAs, (*S*)-reticuline *O*-sulphates, from microbial production were purified by absorption and silica-gel chromatography and analysed to assess their biological activity using a human primary cell-based assay developed to evaluate the biological effects of various drugs by profiling protein biomarkers on human primary cell-based disease models^12,13,18^. A total of 148 biomarkers were measured in 12 types of cultures treated with each BIA (Supplementary Table S4). (*S*)-Reticuline, (*S*)-reticuline 7-*O*-sulphate, and (*S*)-reticuline 3′-*O*-sulphate affected the expression of 47, 30, and 41 biomarkers, respectively, indicating that (*S*)-reticuline and (*S*)-reticuline derivatives showed biological activities (Fig. 3a and Supplementary Table S5).

All of the three BIAs affected 16 biomarkers, including inflammation-related (Fig. 3b), immunomodulatory (Fig. 3c), and tissue-remodelling (Fig. 3d) activities, with similar tendencies observed among the three BIAs. These activities would be derived from the reticuline skeleton, which is a structure common to the three compounds.

The expression of 20 biomarkers was altered in a sulphation-specific way (Fig. 3a). In the BE3C system, which is the model of lung inflammation, intercellular-adhesion molecule-1 (ICAM1) was negatively affected by both sulphated reticulines, but not affected or affected only slightly in the culture treated with (*S*)-reticuline (Fig. 3e). Furthermore, the expression of soluble tumour necrosis factor-α (sTNF-α) in the LPS system was slightly upregulated by (*S*)-reticuline, with the strength of upregulation increased by both sulphated reticulines and indicating that sulphation enhanced the biological activities of the parental BIAs (Fig. 3e). Therefore, sulphate conjugation with BIAs was capable of altering their original activity, which would constitute an effective property for use in drug discovery.

**Figure 4:**
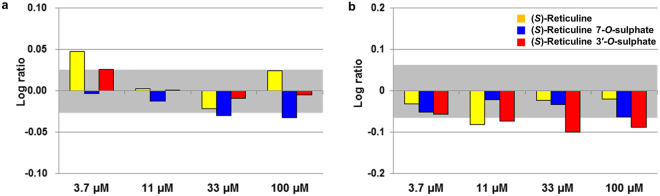
Drug-candidate properties of sulphated (S)-reticulines. (**a**) The Alzheimer’s disease-related biomarker intercellular adhesion molecule-1 (ICAM1) expression in the bronchial disease model BF4T treated with (*S*)-reticuline 7-*O*-sulphate. (**b**) A biomarker, tissue inhibitor of metalloproteinase (TIMP1), involving a non-steroidal anti-inflammatory drug in the fibrosis disease model MyoF treated with (*S*)-reticuline 3′-*O*-sulphate.

The biomarker profiles can be compared with the profile database of reference chemicals (>3,000 agents), enabling prediction of the medical effects of (*S*)-reticuline derivatives. The profile of (*S*)-reticuline was similar to that found in the anti-hypertensive agents amlodipine and methyclothiazide, according to the reference chemical database (Supplementary Table S6a). Because it was reported that (*S*)-reticuline has anti-hypertensive effects dependent upon Ca^2+^-transport inhibition^19^, this human primary cell-based assay could be applicable for deducing the drug efficacy of (*S*)-reticuline derivatives. Comparison of the biomarker profile of (*S*)-reticuline 7-*O*-sulphate with those in the reference drug database suggested that it was similar to that of semagacestat (Supplementary Table S6b), a drug for Alzheimer’s disease. In the bronchial-disease model BF4T, ICAM1 expression was significantly decreased upon treatment with (*S*)-reticuline 7-*O*-sulphate (Fig. 4a). Because ICAM1 appears in senile plaques of patients with Alzheimer’s disease^20^, ICAM1 downregulation implied a possible effect of (*S*)-reticuline 7-*O*-sulphate as a drug for this disease. (*S*)-Reticuline 3′-*O*-sulphate is similar to carprofen, a non-steroidal anti-inflammatory drug (Supplementary Table S6c). (*S*)-Reticuline 3′-*O*-sulphate appreciably repressed tissue inhibitor of metalloproteinase (TIMP1) expression in the MyoF fibrosis-disease model (Fig. 4b). Because fibrosis is induced by TIMP1 overexpression to inhibit matrix metalloproteinases in chronic inflammation cells, TIMP1 downregulation indicated possible anti-inflammatory activity^21^. Interestingly, non-steroidal anti-inflammatory drugs reportedly exhibit additional effects related to reduced risk of developing Alzheimer’s disease^22^. These findings highlighted the potential of novel BIAs as candidate drugs for treating Alzheimer’s and inflammatory diseases.

## Discussion

In this study, we successfully produced large quantities of (*S*)-reticuline, achieving four-fold improvements in yield as compared with the previous system^7^, with the use of TH instead of tyrosinase primarily contributing to this improvement. However, large amount of l-tyrosine remained unreacted, indicating that hydroxylation of l-tyrosine proceeded at lower speeds compared with the production of l-tyrosine (Supplementary Figure S7). In this BIA-production platform, one molecule of BH_4_ was consumed to produce one molecule of l-DOPA; however, if BH_4_ could be regenerated by pterin-4a-carbinolamine dehydratase (PCD) and dihydropteridine reductase (DHPR), productivity of l-DOPA might be improved further. In fact, exogenously-expressed PCD and DHPR are natively functional in *E. coli* for hydroxytyrosol production^23^. Furthermore, more than 1 g/L of dopamine was detected in the cultures prepared for producing (*S*)-reticuline (Supplementary Figure S7), suggesting that monoamine oxidase (MAO) activity was weaker than dopamine productivity. This indicates that there is a room for improvement such as using a more suitable enzyme from an organism other than *Micrococcus luteus*, and protein engineering. Unreacted norlaudanosoline was also detected in the cultures prepared for producing (*S*)-reticuline. If improvement of both tyrosine hydroxylation and monoamine oxidase activities could be accomplished, next limiting-step would be methylation of norlaudanosoline. This could be achieved by using methyltransferases after norlaudanosoline production is improved.

Here, we produced two non-natural BIAs, (*S*)-reticuline 7-*O*-sulphate and (*S*)-reticuline 3′-*O*-sulphate. Although the one-pot culture method successfully produced (*S*)-reticuline 3′-*O*-sulphate from glucose, this method was unsuccessful for (*S*)-reticuline 7-*O*-sulphate production. Because (*S*)-reticuline-biosynthesis intermediates, such as l-tyrosine, l-DOPA, and dopamine, can also be substrates for hSULT1A3^24,25^, the sulphation activity related to (*S*)-reticuline in the *hSULT1A3op*-expressing strain EM459 might be decreased. Additionally, hSULT1A3 is allosterically inhibited by BH_4_, which is artificially produced in EM459^26^ and would result in decreased hSULT1A3 activity. Indeed, (*S*)-reticuline 7-*O*-sulphate was successfully produced from purified (*S*)-reticuline in the absence of both intermediates and BH_4_ (Fig. 2b and Supplementary Fig. S4). Because stepwise culturing bypasses undesirable reactions where downstream enzymes act on upstream intermediates or cofactors, the efficient production of (*S*)-reticuline 7-*O*-sulphate was ultimately achieved in two-step reactions.

Sulphate conjugation often changes the solubility of compounds and is involved in the detoxification of harmful compounds. However, in this study, sulphate conjugation changed the biological effects of (*S*)-reticuline, suggesting that sulphate conjugation is one of the methods necessary for the synthesis of novel non-natural BIAs, thereby extending the spectrum of biological effects. We found that hSULTs exhibited significant activities on other BIAs, including (*R,S*)-norlaudanosoline and (*S*)-scoulerine (Supplementary Table S2). Furthermore, some flavonoids, such as naringenin and quercetin, were also significantly sulphated (Supplementary Table S2). This wide spectrum of hSULT substrate specificity would facilitate the production of various sulphate-conjugated plant secondary metabolites. In principle, this concept could be applied to other conjugations, such as acetylation, methylation, and glycosylation, using appropriate enzymes. These conjugations could also be applied to other BIA subfamilies, such as protoberberine and morphinan alkaloids. The protoberberine skeleton can be produced from (*S*)-reticuline by the addition of the berberine bridge enzyme to a bacterial (*S*)-reticuline-production system, as demonstrated in yeast systems^27–29^. Morphinan alkaloids have also been produced by engineered *E. coli*^10^ and yeast^11^ strains. By combining BIA skeletons with various conjugated groups, an enormous range of non-natural BIAs can be produced as derivatives of natural BIAs, which can further extend the diversity in biological activities.

We demonstrated the high productivity of the *E. coli* (*S*)-reticuline-production system, subsequent production of novel BIAs, and discovery of the biological activities of these compounds. Therefore, this investigation encompassed evaluation of the method from production to product efficacy. The high productivity of the *E. coli* system represents the key property of the current strategy. Although this approach is not yet very popular, it constitutes an effective method for the microbial production of plant secondary metabolites, which will be extensively performed in future. Furthermore, the concepts presented here contribute to drug discovery by facilitating the development of various applications, including the construction of drug-screening libraries.

## Methods

### Experimental design

The current *E. coli* platform for (*S*)-reticuline production was modified to obtain high amounts of (*S*)-reticuline by exchanging RsTYR with dTH. The resulting (*S*)-reticuline producing strain was used to prepare (*S*)-reticuline to produce sulphated (*S*)-reticulines as synthetic BIAs. These novel BIAs were purified by chromatographic techniques and their physiological activities were assessed by a human primary cell–based assay, which had been developed to evaluate the biological effects of various drugs by profiling protein biomarkers in human primary cell–based disease models.

### Plasmids, strains, and culture conditions

*E. coli* DH5α cells were used as the hosts for genetic manipulations. *E. coli* BL21(DE3) cells were used as the hosts for gene expression and BIA production. The l-Tyr-overproducing strain was obtained according to a previous study^7^ and was used as the basis for fermentative production of BIAs. pET-23a and pCDFPL^30^ were used as vectors, with all plasmids and strains used in this study listed in Supplementary Table S1. *E. coli* strains were cultured in 50 mL of modified Terrific broth containing 3% (w/v) glucose as an alternative carbon source to glycerol (TB-G) at 25 °C with shaking following inoculation of the pre-culture in lysogeny broth (LB) medium. The strains were induced by the addition of 0.1 mM isopropyl β-d-1-thiogalactopyranoside (IPTG) at 12 h, if not otherwise specified. Antibiotics were added to the medium in the following concentrations: 50 μg/mL ampicillin, 50 μg/mL chloramphenicol, 25 μg/mL kanamycin, and 100 μg/mL spectinomycin.

### Construction of plasmids for tyrosine hydroxylation

Genes related to tyrosine hydroxylation were synthesised as codon-optimised genes based on the codon usage of *E. coli* using the OptimumGen algorithm (GenScript), with the inclusion of an additional *Nde*I restriction site overlapping the start codon and *Bam*HI or *Eco*RI sites adjacent to the stop codon (Supplementary Fig. S2). These genes were cloned into the respective sites of the pET-23a vector. For BH_4_ biosynthesis, pET-BsMtrAop, pET-ratPTPSop, and pET-ratSPRop were digested with *Bgl*II and *Bam*HI and ligated with appropriate DNA fragments to construct the plasmids pET-PS01, pET-MP01, pET-MS01, and pET-MPS01 (Supplementary Fig. S1b). For the expression of the optimised *dTH2* gene (*dTH2op*), a *Bgl*II-*Eco*RI fragment containing *dTH2op* was inserted into the *Bgl*II-*Mfe*I site of pCDFPL to obtain pCDF-dTH2op02. The co-expression plasmid for dTH2 was constructed by introducing a *Bgl*II-*Bam*HI fragment of pET-MPS01 into the *Bgl*II site of pCDF-dTH2op02.

### Assessment of dTH2-dependent hydroxylation

To use dTH2 for tyrosine hydroxylation in *E. coli*, a BH_4_-producing strain was obtained. Three genes, *BsMtrAop*, *ratPTPSop*, and *ratSPRop*, were introduced into *E. coli* BL21(DE3) using the pET-23a vector under the control of T7 promoters. Additionally, single-gene-deficient variants were constructed to verify the essentiality of these genes (Supplementary Fig. S1b). The resulting strains were cultured and analysed for the production of biopterins by high-performance liquid chromatography (HPLC). Each strain expressing the BH_4_-biosynthesis genes was transformed with the *dTH2op*-expressing plasmid pCDF-dTH2op02, and *dTH2op-*co-expressing strains were obtained (Supplementary Table S1). The *dTH2op-*co-expressing strains were cultured as described, except for the addition of 0.25 mmol l-Tyr at 24 h. After 48 h, cultures were recovered and analysed for l-DOPA formation using HPLC.

### BIA production in a jar fermenter

The BIA-producing strains were cultured in a jar fermenter as described previously^7^. Strains were cultured in TB-G at 25 °C under a controlled pH of 7.2 and 10% oxygen saturation without feeding of glucose solution. For the production of (*S*)-reticuline 3′-*O*-sulphate, 0.1% (w/v) ammonium sulphate was supplied at an interval of 12 h into the culture after 24-h cultivation.

### Analytical procedures

Cultures and reaction mixtures were collected at appropriate intervals and stored at −20 °C until analysis. Aromatic amino acids and biopterin were analysed by a Shimadzu HPLC system equipped with a Discovery HS F5 column (4.6 × 250 mm; Sigma). l-Tyr, l-DOPA, tyramine, and dopamine were monitored by a UV detector, and biopterin was monitored using a fluorometric detector. Solvent A was 10 mM ammonium-formate (pH 3.0), and solvent B was acetonitrile. The column temperature was set to 35 °C. The following gradient of solvent B was used at a flow rate of 0.5 mL/min: 3% to 20% for 25 min, 20% to 100% for 1 min, 100% for an additional 3.5 min, 100% to 3% for 0.5 min, and 3% for an additional 15 min.

BIAs and plant secondary metabolites were measured by a quadrupole MS/MS coupled with an electrospray ionization system consisting of an Agilent 1100 HPLC system and a 3200 QTRAP mass spectrometer (Applied Biosystems). Most of these compounds were detected as positive ions, but sulphate-conjugated variants were detected as negative ions. Products in samples were separated by a TSKgel ODS-80Ts column (4.6 × 250 mm; Tosoh Bioscience) using a mobile phase consisting of solvent A [0.1% (v/v) acetic acid in water] and solvent B [0.1% (v/v) acetic acid in acetonitrile] at 40 °C with a flow rate of 0.5 mL/min. Quinine and quercetin were separated by a solvent A gradient system as follows: 90% (0–5 min), 90% to 10% (5–25 min), and 10% (25–30 min). Other compounds were separated by the isocratic solvent system as follows: (*S*)-reticuline, 75% solvent A; (*R*,*S*)-norlaudanosoline, morphine, and codeine, 90% solvent A; (*S*)-scoulerine, 80% solvent A; naringenin, 60% solvent A; and rutin, 75% solvent A. The monitored ions were as follows: *m*/*z* = 330 [(*S*)-reticuline], 288 [(*R*,*S*)-norlaudanosoline], 286 (morphine), 300 (codeine), 328 [(*S*)-scoulerine], 325 (quinine), 303 (quercetin), 273 (naringenin), and 611 (rutin).

To identify the positions of the sulphonyl-group substitutions in (*S*)-reticuline *O*-sulphate, the samples were analysed by LC-MS/MS, monitoring a positive ion of *m*/*z* = 410 at 50 V of collision energy (CE) and a negative ion of *m*/*z* = 408 at −50 V of CE.

### Identification of (*S*)-reticuline *O*-sulphate

According to positive-ion analysis by LC-MS/MS, the peak pattern of (*S*)-reticuline *O*-sulphate was almost same as that of (*S*)-reticuline (Supplementary Fig. S4), indicating that daughter ions containing sulphate groups cannot be detected in positive-ion mode. In negative-ion mode analysis of (*S*)-reticuline at *m*/*z* = 328, the daughter ions, *m*/*z* = 121 and 176, were observed, corresponding to benzyl groups and an isoquinoline skeleton lacking a methyl group, respectively. In the analysis of cultures of the *hSULT1A3op*-expressing strain, a specific peak at *m*/*z* = 256 (176 + 80) was found, corresponding to a sulphate-group (+80 Da) addition to the isoquinoline moiety, indicating that this compound was (*S*)-reticuline 7-*O*-sulphate. However, in the cultures of *hSULT1E1op*-expressing strains, the daughter ion at *m*/*z* = 201 (121 + 80) was detected, suggesting that the benzyl group was sulphated. Therefore, we concluded that this compound was (*S*)-reticuline 3′-*O*-sulphate.

### Purification of (*S*)-reticuline from culture medium

Cultures of the (*S*)-reticuline-producing strain EM353 cultured in a jar fermenter for 96 h were centrifuged, and the supernatant was collected. The supernatant was subjected to Diaion HP20 column chromatography (20 × 250 mm; Sigma-Aldrich) and eluted with a stepwise gradient of H_2_O (solvent A) and 0.1% ammonium in methanol (solvent B): 0% B, 90 mL; 60% B, 180 mL; and 100% B, 180 mL. The fraction of 100% B elution, which contained most of the (*S*)-reticuline, was diluted with an equal volume of water and subjected to Diaion HP20 column chromatography (10 × 250 mm). This fraction was then eluted with a linear gradient of solvents A and B at a flow rate of 1.0 mL/min as follows: 50% to 100% solvent B (0–120 min) and 100% solvent B (120–180 min). The fractions containing (*S*)-reticuline were collected, and the partially pure fractions were applied to Silicagel 60 (Nacalai Tesque) column chromatography and developed with chloroform:methanol (17:3). The pure fractions of (*S*)-reticuline were collected and concentrated for other applications.

### Sulphate conjugation of plant secondary metabolites using *hSULT*-expressing *E. coli*

The genes *hSULT1A1*, *hSULT1A3*, and *hSULT1E1* were synthesised as codon-optimised genes as previously described (Supplementary Fig. S2). The synthetic genes *hSULT1A1op*, *hSULT1A3op*, and *hSUlT1E1op* were cloned in *Nde*I and *Bam*HI sites of pET-23a to obtain the plasmids pET-hSULT1A1op, pET-hSULT1A3op, and pET-hSULT1E1op, respectively. These plasmids were introduced into *E. coli* BL21(DE3) cells, and the *hSULT*-expressing *E. coli* strains were cultured in 50 mL TB-G at 25 °C with shaking after inoculation of the pre-culture in LB medium. After induction with IPTG at 12 h, 5 mL of these cultures was separated into other flasks containing 0.1 mM of plant secondary metabolites, 100 mM 2-morpholinoethanesulphonic acid, and 0.2% (w/v) ammonium sulphate and incubated for 12 h at 25 °C. Sulphate conjugations were determined by LC-MS as the decrease in substrates compared to that in the control strain (AN783).

### Preparation of (*S*)-reticuline *O*-sulphates

Purified (*S*)-reticuline was used as the substrate for biotransformation with *E. coli* transformants to produce (*S*)-reticuline *O*-sulphates. *E. coli* BL21(DE3) strains expressing *hSULTs* were cultivated as described, and 10 μmol of purified (*S*)-reticuline was supplied at the time of induction. Ammonium sulphates were added to the cultures at a final concentration of 0.2% (w/v) for the 12-h and 36-h incubations. The 60-h cultures were recovered, and the supernatants were analysed for (*S*)-reticuline *O*-sulphates by LC-MS. Each of these supernatants was subjected to Diaion HP20 column chromatography (10 × 250 mm) and eluted with a linear gradient of solvents A and B at a flow rate of 1.0 mL/min as follows: 0% to100% solvent B (0–100 min) and 100% solvent B (100–120 min). Fractions containing the desired (*S*)-reticuline *O*-sulphates were collected and subjected to Silicagel 60 column chromatography (10 × 150 mm) equilibrated with chloroform:methanol solution. The ratios of chloroform:methanol used were 7:3 for (*S*)-reticuline 7-*O*-sulphate and 8:2 for (*S*)-reticuline 3′-*O*-sulphate. The pure fractions of (*S*)-reticuline *O*-sulphates were collected and concentrated.

### Prediction of the biological activities of (*S*)-reticuline derivatives by human primary cell-based assay

Three compounds synthesised in the current study, including two novel BIA (*S*)-reticuline *O*-sulphates, were predicted to have biological roles in humans by assay systems employing human primary cell disease models (BioMAP; DiscoveRx) (Supplementary Table S4). Compounds were dissolved in dimethyl sulfoxide as a 100-μM solution and prepared for 3-, 9-, and 27-fold diluted solutions using serial dilutions. These solutions were used for assays against the Diversity PLUS panels (DiscoveRx) consisting of 12 human-disease models as previously described^31^. When biomarker expression was significantly changed in more than two of four concentrations with the same tendency (positively or negatively), we judged that the biomarker was affected by the (*S*)-reticuline derivative. Although some biomarkers were affected with opposite tendencies, both positively and negatively, by certain compounds, we could not eliminate the possibility of experimental errors from these results; therefore, we did not include this data. Omitted data were indicated in Supplementary Table S5 as ‘Amb’. By measuring biomarker readouts in the BioMAP systems, the resulting activity profiles were compared to the profiles of reference compounds in the BioMAP database comprising over 3000 compounds.

### Production of (*S*)-reticuline *O*-sulphates from glucose

To incorporate the *hSULT* genes in the (*S*)-reticuline–producing strain EM353, the l-Tyr-hydroxylating plasmid pCDF-MPSTd02 was reconstructed for co-expression with the *hSULT1A3op* or *hSULT1E1op* genes. Plasmids pET-hSUL1A3op and pET-hSULT1E1 were digested with *Bam*HI and *Bgl*II, and the resulting fragments containing *hSULTs* were ligated into *Bgl*II-digested pCDF-MPSTd02. The resulting plasmids containing the target genes *hSULT1A3op* or *hSULT1E1op* in the same direction were designated as pCDF-S1A3MPSTd02 and pCDF-S1E1MPSTd02, respectively (Supplementary Table S1). On the basis of the strain EM353, *E. coli* BL21(DE3)*ΔtyrR* was transformed with four plasmids bearing pCDF-S1A3MPSTd02 or pCDF-S1E1MPSTd02 instead of pCDF-MPSTd02. The resulting strains, EM459 and EM437, were grown on TB-G medium supplemented with ammonium sulphate at a final concentration of 0.1% (w/v) every 24-h post-induction. Cultures were collected at appropriate intervals and analysed for (*S*)-reticuline production. For large-scale fermentation of (*S*)-reticuline 3′-*O*-sulphate, strain EM437 was cultured in a 3-L jar fermenter, as previously described, and was supplemented with 0.1% (w/v) ammonium sulphate every 12 h after 24-h incubation.

## Electronic supplementary material

Supplementary information

Supplementary Table S5

## References

[1] Newman DJ, Cragg GM (2012). Natural products as sources of new drugs over the 30 years from 1981 to 2010. J. Nat. Prod..

[2] Song MC (2014). Microbial biosynthesis of medicinally important plant secondary metabolites. Nat. Prod. Rep..

[3] Peplow M (2016). Synthetic biology’s first malaria drug meets market resistance. Nature.

[4] Qian P, Yang X-W (2014). Five new alkaloids from Coptidis Rhizoma–Euodiae Fructus couple and their cytotoxic activities against gastrointestinal cancer cells. Fitoterapia.

[5] Lv J-J (2013). Cytotoxic bisbenzylisoquinoline alkaloids from *Stephania epigaea*. J. Nat. Prod..

[6] Ziegler J, Facchini PJ (2008). Alkaloid biosynthesis: metabolism and trafficking. Annu. Rev. Plant Biol..

[7] Nakagawa A (2011). A bacterial platform for fermentative production of plant alkaloids. Nat. Commun..

[8] DeLoache WC (2015). An enzyme-coupled biosensor enables (*S*)-reticuline production in yeast from glucose. Nat. Chem. Biol..

[9] Trenchard IJ, Siddiqui MS, Thodey K, Smolke CD (2015). *De novo* production of the key branch point benzylisoquinoline alkaloid reticuline in yeast. Metab. Eng..

[10] Nakagawa A (2016). Total biosynthesis of opiates by stepwise fermentation using engineered *Escherichia coli*. Nat. Commun..

[11] Galanie S, Thodey K, Trenchard IJ, Filsinger Interrante M, Smolke CD (2015). Complete biosynthesis of opioids in yeast. Science.

[12] Berg EL (2010). Chemical target and pathway toxicity mechanisms defined in primary human cell systems. J. Pharmacol. Toxicol. Methods.

[13] Berg EL, Hsu Y-C, Lee JA (2014). Consideration of the cellular microenvironment: physiologically relevant co-culture systems in drug discovery. Adv. Drug Deliv. Rev..

[14] Hernández-Romero D, Sanchez-Amat A, Solano F (2006). A tyrosinase with an abnormally high tyrosine hydroxylase/dopa oxidase ratio. FEBS J..

[15] Pribat A (2010). FolX and FolM are essential for tetrahydromonapterin synthesis in *Escherichia coli* and *Pseudomonas aeruginosa*. J. Bacteriol..

[16] Yamamoto K (2003). Genetic engineering of *Escherichia coli* for production of tetrahydrobiopterin. Metab. Eng..

[17] Wang L-Q, James MO (2006). Inhibition of sulfotransferases by xenobiotics. Curr. Drug Metab..

[18] Berg EL, Yang J, Polokoff MA (2013). Building predictive models for mechanism-of-action classification from phenotypic assay data sets. J. Biomol. Screen..

[19] Martin ML (1993). Antispasmodic activity of benzylisoquinoline alkaloids analogous to papaverine. Planta Med..

[20] Akiyama H (1993). Expression of intercellular adhesion molecule (ICAM)−1 by a subset of astrocytes in Alzheimer disease and some other degenerative neurological disorders. Acta Neuropathol..

[21] Hu J, V den Steen PE, Sang Q-XA, Opdenakker G (2007). Matrix metalloproteinase inhibitors as therapy for inflammatory and vascular diseases. Nat. Rev. Drug Discov..

[22] Rubio-Perez JM, Morillas-Ruiz JM (2012). A review: inflammatory process in Alzheimer’s disease, role of cytokines. Sci. World J..

[23] Satoh Y, Tajima K, Munekata M, Keasling JD, Lee TS (2012). Engineering of l-tyrosine oxidation in *Escherichia coli* and microbial production of hydroxytyrosol. Metab. Eng..

[24] Sakakibara Y (2003). Manganese-dependent Dopa/tyrosine sulfation in HepG2 human hepatoma cells: novel Dopa/tyrosine sulfotransferase activities associated with the human monoamine-form phenol sulfotransferase. Biochim. Biophys. Acta.

[25] Dajani R, Hood AM, Coughtrie MWH (1998). A single amino acid, Glu146, governs the substrate specificity of a human dopamine sulfotransferase, SULT1A3. Mol. Pharmacol..

[26] Cook I, Wang T, Leyh TS (2017). Tetrahydrobiopterin regulates monoamine neurotransmitter sulfonation. Proc. Natl. Acad. Sci. USA.

[27] Fossati E (2014). Reconstitution of a 10-gene pathway for synthesis of the plant alkaloid dihydrosanguinarine in *Saccharomyces cerevisiae*. Nat. Commun..

[28] Hori K, Okano S, Sato F (2016). Efficient microbial production of stylopine using a *Pichia pastoris* expression system. Sci. Rep..

[29] Trenchard IJ, Smolke CD (2015). Engineering strategies for the fermentative production of plant alkaloids in yeast. Metab. Eng..

[30] Kim J-S (2013). Improvement of reticuline productivity from dopamine by using engineered *Escherichia coli*. Biosci. Biotechnol. Biochem..

[31] Haselmayer P (2014). Characterization of novel PI3Kδ inhibitors as potential therapeutics for SLE and lupus nephritis in pre-clinical studies. Front. Immunol..

