# Supplementary material for: Microbial production of novel sulphated alkaloids for drug discovery
Source: Sci Rep. 2018 May 22;8:7980. doi: 10.1038/s41598-018-26306-7 (PMC5964154; doi:10.1038/s41598-018-26306-7)
Supplement: Supplementary file 1 — Supplementary information [file 41598_2018_26306_MOESM1_ESM.docx]

Microbial production of novel sulphated alkaloids for drug discovery

**Eitaro Matsumura^1,+^, Akira Nakagawa^1,+^, Yusuke Tomabechi^2^, Shinichi Ikushiro^3^, Toshiyuki Sakaki^3^, Takane Katayama^4^, Kenji Yamamoto^1^, Hidehiko Kumagai^1^, Fumihiko Sato^4^, and Hiromichi Minami^1,*^**

^1^Research Institute for Bioresources and Biotechnology, Ishikawa Prefectural University, 1-308 Suematsu, Nonoichi, Ishikawa 921-8836, Japan.

^2^Department of Applied Chemistry, School of Engineering, Tokai University, 4-1-1 Kitakaname, Hiratsuka, Kanagawa 259-1292, Japan.

^3^Department of Biotechnology, Faculty of Engineering, Toyama Prefectural University, 5180 Kurokawa, Imizu, Toyama 939-0398, Japan.

^4^Division of Integrated Life Science, Graduate School of Biostudies, Kyoto University, Sakyo-ku, Kyoto 606-8502, Japan.

^*^Corresponding author. Email: [minami@ishikawa-pu.ac.jp](mailto:minami@ishikawa-pu.ac.jp)

^+^These authors contributed equally to this work.

**Supplementary information**

**
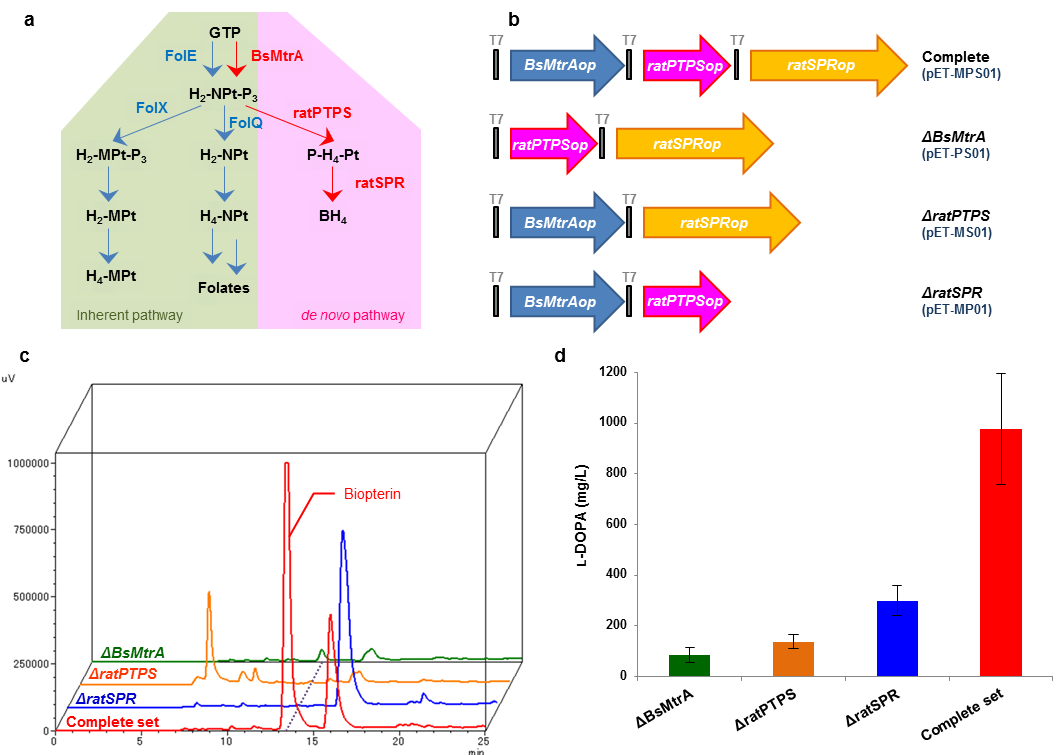
**

**Supplementary Figure S1. BH_4_-dependent hydroxylation catalysed by dTH2 in *Escherichia* *coli*.** (**a**) GTP is converted into three kinds of pterins, with dihydroneopterin triphosphate (H_2_-NPt-P_3_) as the branching point. FolE and BsMtrA are GTP cyclohydrolases. FolE, FolX (dihydroneopterin triphosphate 2′-epimerase), and FolQ (dihydroneopterin triphosphate pyrophosphohydrolase) are inherent enzymes producing naturally occurring pterin-tetrahydroneopterin (H_4_-NPt) and tetrahydromonapterin (H_4_-MPt). BsMtrA, ratPTPS, and ratSPR are heterogeneous enzymes for BH_4_ production and were overexpressed in *E. coli.* The abbreviations are as follows: P-H_4_-Pt, 6-pyruvoyltetrahydropterin; H_2_-MPt-P_3_, dihydromonapterin triphosphate; H_2_-MPt, dihydromonapterin; H_2_-NPt, dihydroneopterin. (**b**) Construction of plasmids for the expression of BH_4_-biosynthesis genes. (**c**) Fluorometric high-performance liquid chromatography profiles of biopterins from *E. coli* strains expressing the BH_4_-biosynthesis genes and detected as the oxidised form biopterin. (**d**) l-Tyr hydroxylation of dTH2 using the combination of BH_4_-biosynthetic genes. Strains were grown in Terrific broth containing glucose (TB-G) at 25°C with shaking and were induced with 0.1 mM IPTG at 12 h. For l-DOPA production, 5 mM l-Tyr was added after 24*-*h incubation. The strain harbouring the complete set of BH_4_-biosynthesis enzymes is indicated as complete; single-gene-deficient variants are indicated as Δ-prefixed enzyme names.


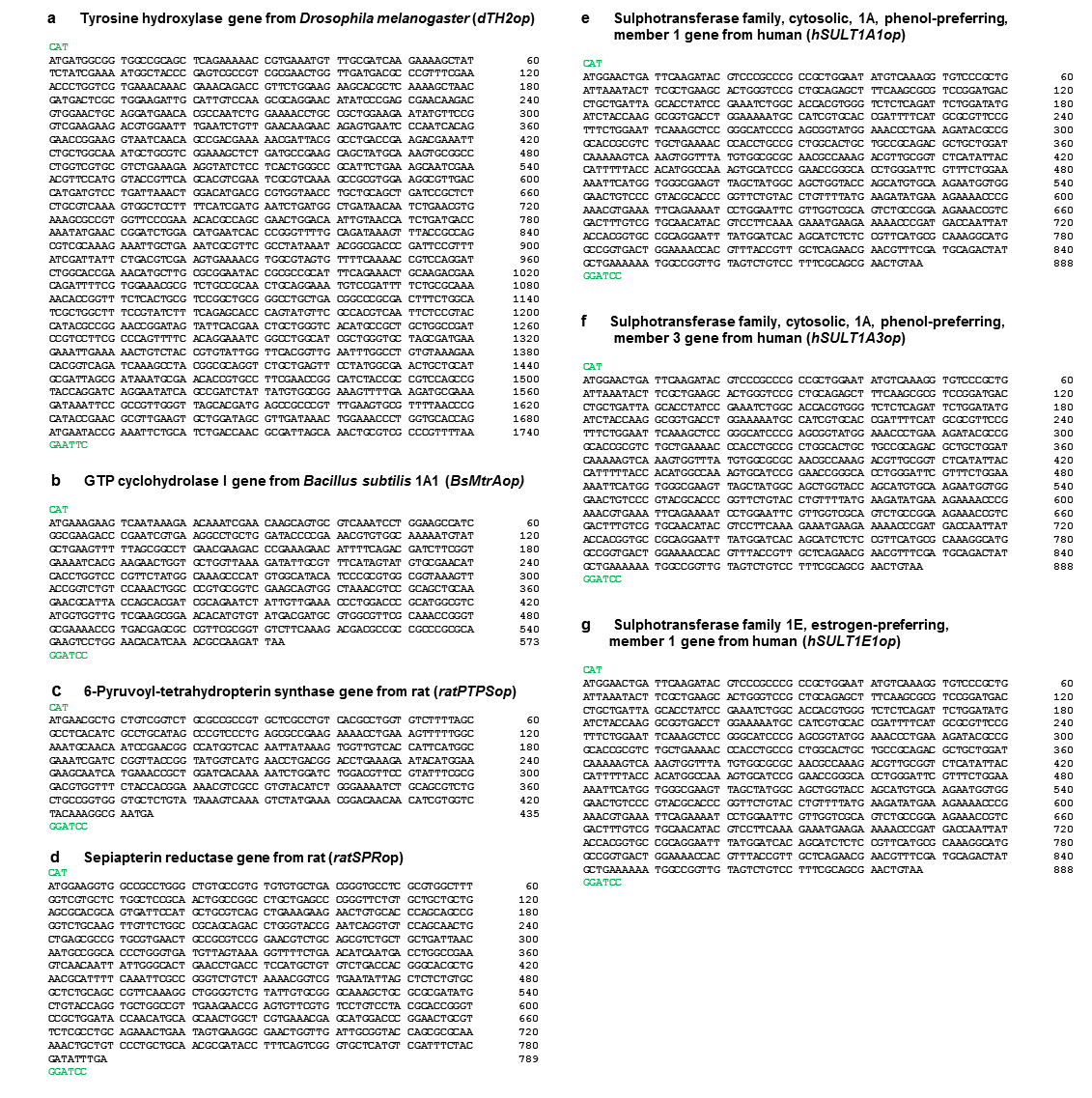


**Supplementary Figure S2. Nucleotide sequences of optimised genes for expression in *Escherichia coli*.**

**
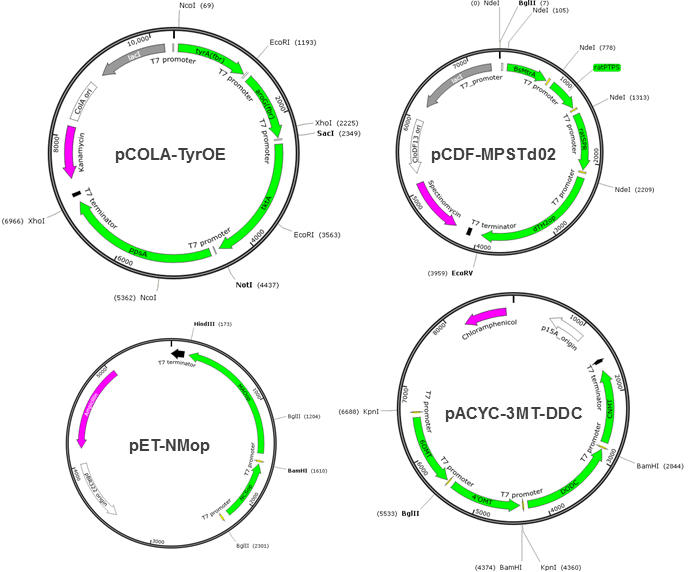
**

**Supplementary Figure S3. Plasmid maps for fermentative production of (*S*)-reticuline.**


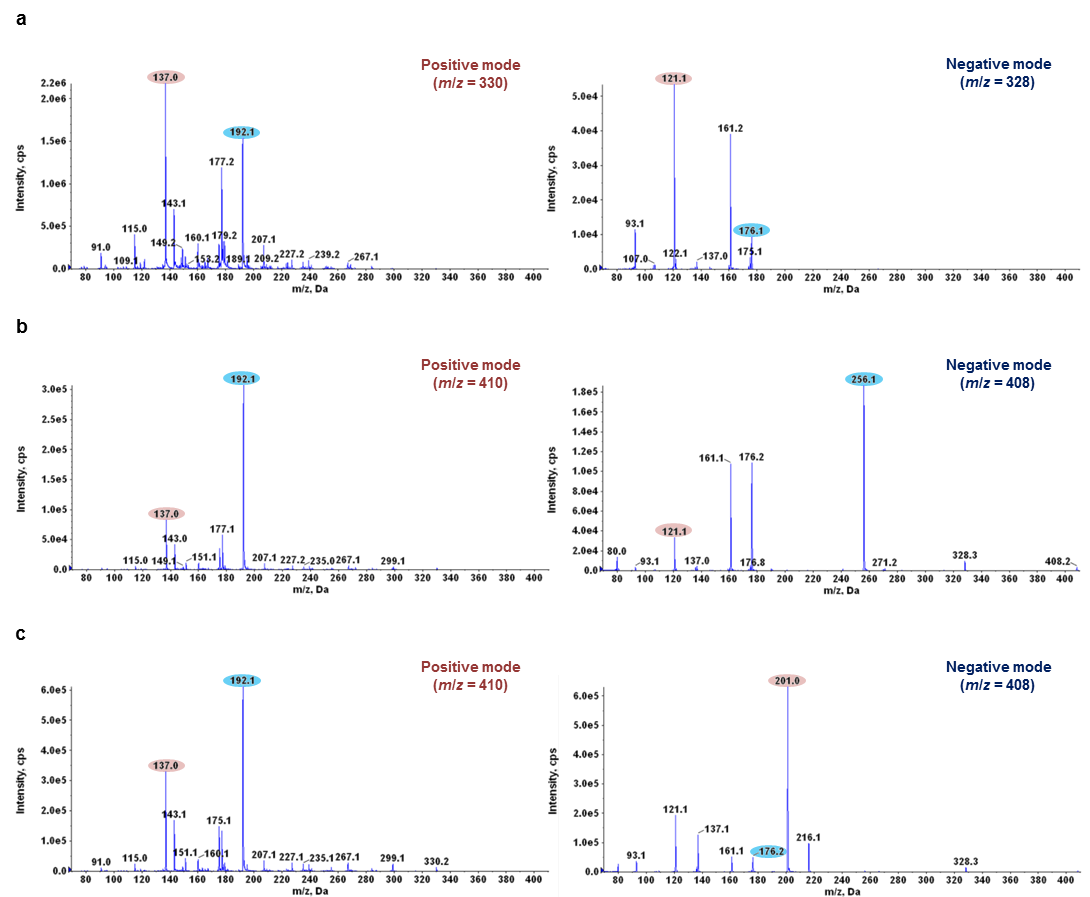


**Supplementary Figure S4. Analysis of (*S*)-reticuline *O*-sulphates by LC-MS/MS.** (**a–c**) Product-ion mass spectra of (*S*)-reticuline (a), (*S*)-reticuline 7-*O*-sulphate (b), and (*S*)-reticuline 3′-*O*-sulphate (c). (*S*)-Reticuline *O*-sulphates were analysed for the positive ion of *m*/*z* = 410 and the negative ion of *m*/z = 408 in LC-MS/MS, whereas (*S*)-reticuline was represented by the positive ion of *m*/*z* = 330 and the negative ion of *m*/*z* = 328. Red and blue ovals indicate the daughter ion originating from the benzyl group and isoquinoline moiety, respectively.


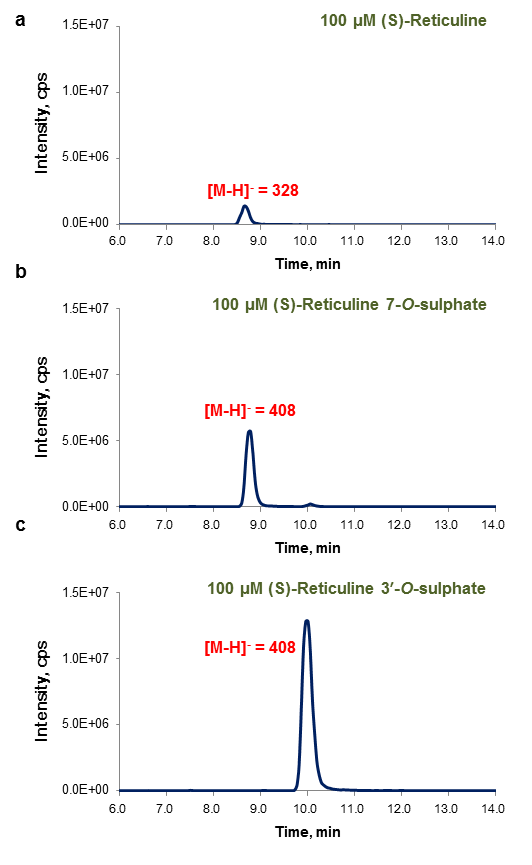


**Supplementary Figure S5. Intensity of negative ions of (*S*)-reticuline (a), (*S*)-reticuline 7-*O*-sulphate (b), and (*S*)-reticuline 3′-*O*-sulphate (c) at a concentration of 100 μM in LC-MS analysis.** (*S*)-Reticuline *O*-sulphates were analysed for the negative ion of *m*/z = 408 in LC-MS, whereas (*S*)-reticuline was represented by the negative ion of *m*/*z* = 328.


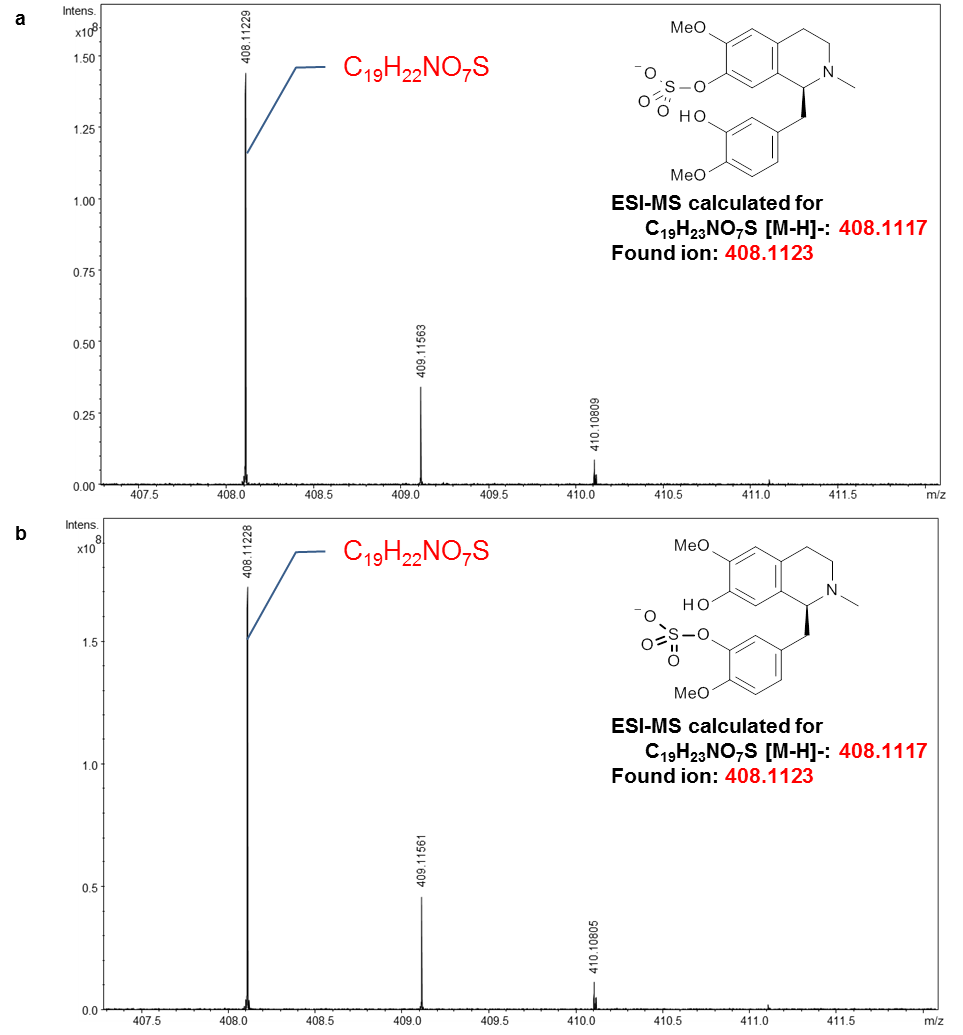


**Supplementary Figure S6.** High-resolution exact-mass spectra of (*S*)-reticuline 7-*O*-sulphate **(a)** and (*S*)-reticuline 3′-O-sulphate **(b)** in the ESI negative ion mode obtained by using Fourier transform ion cyclotron resonance mass spectrometer.

**Supplementary Figure S7.**
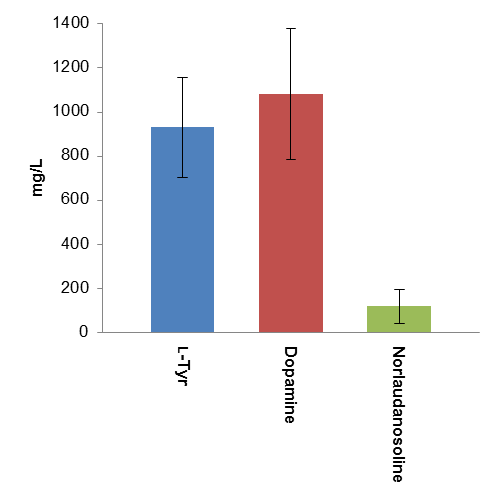
 **Residual intermediates in (*S*)-reticuline producing EM353 culture.**

**Supplementary Table S1. Plasmids and strains.**

(**a**) Plasmids

| Plasmids | Description | Source |
| --- | --- | --- |
| pET-23a | Ampicillin-resistant, expression vector for *E. coli* | Novagen |
| pCDFPL | Spectinomycin resistant, expression vector for *E. coli* | 30 |
| pET-MPS01 | *BsMtrAop*, *ratPTPSop* and *ratSPRop* in pET-23a | This study |
| pET-PS01 | *ratPTPSop* and *ratSPRop* in pET-23a | This study |
| pET-MS01 | *BsMtrAop* and *ratSPRop* in pET-23a | This study |
| pET-MP01 | *BsMtrAop* and *ratPTPSop* in pET-23a | This study |
| pCDF-dTH2op02 | *dTH2op* in pCDFPL | This study |
| pCDF-MPSTd02 | *BsMtrAop*, *ratPTPSop*, *ratSPRop*, and *dTH2op* in pCDFPL | This study, Supplementary Fig. S3 |
| pACYC-3MT | *6OMT*, *CNMT*, and *4′OMT* in pACYC184 | 32 |
| pACYC-3MT-DDC | *6OMT*, *CNMT*, *DODC*, and *4′OMT* in pACYC184 | This study, Supplementary Fig. S3 |
| pCOLA-TyrOE | *tyrA^fbr^*, *aroG^fbr^*, *tktA*, and *ppsA* in pCOLADuet-1 | 7, Supplementary Fig. S3 |
| pET-NMop | *NCSop* and *MAOop* in pET-23a | This study, Supplementary Fig. S3 |
| pET-NDMop | *NCSop*, *DODC* and *MAOop* in pET-23a | This study |
| pET-hSULT1A1op | *hSULT1A1op* in pET-23a | This study |
| pET-hSULT1A3op | *hSULT1A3op* in pET-23a | This study |
| pET-hSULT1E1op | *hSULT1E1op* in pET-23a | This study |
| pCDF-S1A3MPTd02 | *hSULT1A3op*, *BsMtrAop*, *ratPTPSop*, *ratSPRop* and *dTH2op* in pCDFPL | This study |
| pCDF-S1E1MPTd02 | *hSULT1E1op*, *BsMtrAop*, *ratPTPSop*, *ratSPRop* and *dTH2op* in pCDFPL | This study |

(**b**) Strains

| Strains | Description | Source |
| --- | --- | --- |
| *E. coli* DH5α | Native strain | Novagen |
| *E. coli* BL21(DE3) | Native strain | Novagen |
| *E. coli* BL21(DE3)Δ*tyrR* | *tyrR*-deleted mutant strain of *E. coli* BL21(DE3) | 7 |
| AN783 | pET-23a in *E. coli* BL21(DE3) | This study |
| EM495 | pET-PS01 in *E. coli* BL21(DE3) | This study |
| EM496 | pET-MS01 in *E. coli* BL21(DE3) | This study |
| EM497 | pET-MP01 in *E. coli* BL21(DE3) | This study |
| EM498 | pET-MPS01 in *E. coli* BL21(DE3) | This study |
| EM499 | pET-PS01 and pCDF-dTH2op02 in *E. coli* BL21(DE3) | This study |
| EM500 | pET-MS01 and pCDF-dTH2op02 in *E. coli* BL21(DE3) | This study |
| EM501 | pET-MP01 and pCDF-dTH2op02 in *E. coli* BL21(DE3) | This study |
| EM502 | pET-MPS01 and pCDF-dTH2op02 in *E. coli* BL21(DE3) | This study |
| EM332 | pCDF-MPSTd02 in *E. coli* BL21(DE3) | This study |
| EM375 | pCDF-RsTYR in *E. coli* BL21(DE3) | This study |
| EM494 | pCDF-MPSTd02, pET-NDMop, and pACYC-3MT in *E. coli* BL21(DE3) | This study |
| EM308 | pCDF-RsTYR, pET-NDMop, and pACYC-3MT in *E. coli* BL21(DE3) | This study |
| EM353 | pCOLA-TyrOE, pET-NMop, pCDF-MPSTd02, and pACYC-3MT-DDC in *E. coli* BL21(DE3)Δ*tyrR* | This study |
| EM406 | pET-hSULT1A1op in *E. coli* BL21(DE3) | This study |
| EM407 | pET-hSULT1A3op in *E. coli* BL21(DE3) | This study |
| EM408 | pET-hSULT1E1op in *E. coli* BL21(DE3) | This study |
| EM459 | pCOLA-TyrOE, pET-NMop, pCDF-S1A3MPSTd02, and pACYC-3MT-DDC in *E. coli* BL21(DE3)Δ*tyrR* | This study |
| EM437 | pCOLA-TyrOE, pET-NMop, pCDF-S1E1MPSTd02, and pACYC-3MT-DDC in *E. coli* BL21(DE3)Δ*tyrR* | This study |

**Supplementary Table S2. Sulphate conjugation of plant secondary metabolites in *hSULT*-expressing *Escherichia coli.***

| Compound | Sulphate conjugation (% of substrate) | | |
| --- | --- | --- | --- |
|  | EM406 (hSULT1A1) | EM407 (hSULT1A3) | EM408 (hSULT1E1) |
| Dopamine | ND | 8.12 ±1.60 | ND |
| (*R*,*S*)-Norlaudanosoline | ND | 7.50 ± 2.47 | ND |
| (*S*)-Reticuline | ND | 31.9 ± 1.3 | 58.5 ± 2.1 |
| Morphine | ND | ND | ND |
| Codeine | ND | ND | ND |
| (*S*)-Scoulerine | 1.41 ± 0.24 | 30.7 ± 4.2 | 24.1 ± 1.5 |
| Quinine | ND | ND | ND |
| Quercetin | ND | 42.7 ± 1.6 | 44.5 ± 3.4 |
| Naringenin | 7.9 ± 2.1 | 65.6 ± 8.1 | 60.8 ± 5.3 |
| Rutin | ND | ND | 1.44 ± 0.51 |

ND: not detected.

**Supplementary Table S3. Production of (*S*)-reticuline *O*-sulphates with *hSULT*-expressing *Escherichia coli* from purified (*S*)-reticuline.**

| Strains | Production (mg/L) | | Selectivity of hydroxyl group |
| --- | --- | --- | --- |
| (hSULTs) | (*S*)-Reticuline 7-*O*-sulphate | (*S*)-Reticuline 3′-*O*-sulphate |  |
| EM407 (hSULT1A3) | 55.8 ± 2.1 | 9.5 ± 0.5 | 85.5% for 7-OH group |
| EM408 (hSULT1E1) | 5.0 ± 0.1 | 114.8 ± 4.3 | 95.8% for 3′-OH group |

**Supplementary Table S4. Physiologically relevant models of human disease states.**

| System | Primary human cell types | Cytokines/activators | Disease/tissue relevance | Readout parameters |
| --- | --- | --- | --- | --- |
| 3C | Venular endothelial cells | IL1B TNFα IFNG | Cardiovascular disease Chronic inflammation | CAM1, VCAM1, ICAM1, PLAUR, SELE,CCL2, CXC9, HLA-DR, IL8, THBD, F3~~,~~ proliferation, total protein levels measured by SRB staining (SRB) |
| 4H | Venular endothelial cells | IL4 Histamine | Asthma Allergy Oncology | CCL2, CCL26, VCAM1, SELP, PLAUR, KDR, SRB |
| LPS | Peripheral blood mononuclear cells Endothelial cells | TLR4 | Cardiovascular disease Chronic inflammation | CCL2, VCAM1, THBD, F3, CD40, SELE, CD69, CXCL8, IL1A, CSF1, sPGE2, sTNF-α~~,~~ SRB |
| SAg | Peripheral blood mononuclear cells Endothelial cells | T\-cell receptor (TCR) | Autoimmune disease Chronic inflammation | CCL2, CD38, CD40, SELE, CD69, CXCL8, CXCL9, peripheral blood mononuclear cell (PBMC) cytotoxicity, SRB, proliferation |
| BT | B cells Peripheral blood mononuclear cells | anti-IgM TCR | Asthma Allergy Oncology Autoimmunity | B cell proliferation, PBMC cytotoxicity, sIL2, sTNF-α, sIL6, secreted IgG, sIL17A, sIL17F |
| BE3C | Bronchial epithelial cells | IL1B TNFα IFNG | COPD Lung | ICAM1,PLAUR, CXCL8, CXCL9, CXCL10, CXCL11, EGFR, HLA-DR, IL1A, KRT8/KRT18, MMP1, MMP9, SERPINE1, SRB, PLAT, PLAU |
| BF4T | Bronchial epithelial cells Fibroblasts | TNFα IL4 | Asthma Allergy Fibrosis Lung | CCL2, CCL26, VCAM1, ICAM1, CD90, CXCL8, IL1A, KRT8/KRT18, MMP1, MMP3, MMP9, SERPINE1, SRB, PLAT, PLAU |
| HDF3CGF | Fibroblasts | IL1B TNFα IFNG EGF FGF2 PDGF-BB | Fibrosis Chronic inflammation | CCL2, VCAM1, ICAM1, CXCL8, CXCL9, CXCL10, CXCL11, collagen I, collagen III, EGFR, CSF1, SERPINE1, MMP1, proliferation (72 h), TIMP1, TIMP2, SRB |
| KF3CT | Keratinocytes Fibroblasts | IL1B TNFα IFNG TGFβ | Psoriasis Dermatitis Skin | CCL2, ICAM1, CXCL8, CXCL9, CXCL10, IL1A, MMP9, SERPINE1, TIMP2, PLAU, SRB |
| CASM3C | Coronary artery smooth muscle cells | IL1B TNFα IFNG | Cardiovascular inflammation Restenosis | CCL2, VCAM1, THBD, F3, PLAUR, CXCL8, CXCL9, HLA-DR, IL6, LDLR, CSF1, SERPINE1, serum amyloid A, SRB, proliferation |
| MyoF | Lung fibroblasts | TNFα TGFβ | Fibrosis Chronic inflammation | FGF2, VCAM1, CXCL8, collagen I, collagen III, collagen IV, DCN~~,~~ α-smooth muscle (SM) actin, TIMP1, SRB, MMP1, SERPINE1 |
| /Mphg | Venular endothelial cells M1 macrophages | TLR2 | Cardiovascular Inflammation Restenosis Chronic inflammation | CCL2, CCL3, VCAM1, CD40, SELE, CD69, CXCL8, IL1A, CSF1, sIL10, SRB, SRB-Mphg |

**Supplementary Table S6. BioMAP-database matching of (*S*)-reticuline derivatives.**

(**a**) (*S*)-Reticuline

| Concentration | Database match | Z-score | Pearson’s  score | Database match  mechanism class |
| --- | --- | --- | --- | --- |
| 100 μM | Amlodipine (3.3 μM) | 7.495 | 0.568 | Calcium channel blocker |
|  | FICZ (1 μM) | 6.825 | 0.528 | AhR agonist |
|  | FICZ (330 nM) | 6.78 | 0.525 | AhR agonist |
| 33 μM | Amlodipine (3.3 μM) | 7.349 | 0.56 | Calcium channel blocker |
|  | Benzoic acid (10 μM) | 5.723 | 0.456 | Anti-fungal agent |
|  | Methyclothiazide (1.1 μM) | 5.488 | 0.44 | Diuretic, anti-hypertensive |
| 11 μM | Amlodipine (1.1 μM) | 8.76 | 0.637 | Calcium channel blocker |
|  | Methyclothiazide (1.1 μM) | 8.527 | 0.625 | Diuretic, anti-hypertensive |
|  | Aminosalicylate sodium (1.1 μM) | 7.274 | 0.555 | Anti-tubercular agent |
| 3.7 μM | Methyclothiazide (1.1 μM) | 11.781 | 0.767 | Diuretic, anti-hypertensive |
|  | Amlodipine (1.1 μM) | 10.662 | 0.725 | Calcium channel blocker |
|  | Aminosalicylate sodium (1.1 μM) | 9.518 | 0.675 | Anti-tubercular agent |

(**b**) (*S*)-Reticuline 7-*O*-sulphate

| Concentration | Database match | Z-score | Pearson’s  score | Database match  mechanism class |
| --- | --- | --- | --- | --- |
| 100 μM | Semagacestat (3.3 μM | 7.382 | 0.562 | γ-Secretase inhibitor |
|  | Semagacestat (10 μM) | 7.174 | 0.549 | γ-Secretase inhibitor |
|  | Abiraterone acetate (3.3 μM) | 7.066 | 0.543 | 17α-hydroxylase/C17,20 lyase inhibitor |
| 33 μM | Aminosalicylate sodium (10 μM) | 9.043 | 0.652 | Anti-tubercular agent |
|  | Betaxolol (3.3 μM) | 7.328 | 0.559 | Beta1 receptor blocker |
|  | Tropicamide (10 μM) | 7.031 | 0.541 | Anti-muscarinic agent |
| 11 μM | Aminosalicylate sodium (3.3 μM) | 9.831 | 0.689 | Anti-tubercular agent |
|  | Betaxolol (3.3 μM) | 8.707 | 0.635 | Beta1 receptor blocker |
|  | Benzoic acid (1.1 μM) | 8.004 | 0.597 | Anti-fungal agent |
| 3.7 μM | Oxymetholone (1.1 μM) | 9.682 | 0.682 | Synthetic anabolic steroid |
|  | Aminosalicylate sodium (1.1 μM) | 9.344 | 0.666 | Anti-tubercular agent |
|  | Tropicamide (1.1 μM) | 9.202 | 0.660 | Anti-muscarinic agent |

(**c**) (*S*)-Reticuline 3′-*O*-sulphate

| Concentration | Database match | Z-score | Pearson’s  score | Database match  mechanism class |
| --- | --- | --- | --- | --- |
| 100 μM | Valaciclovir HCl (1.1 μM) | 7.007 | 0.539 | Viral DNA polymerase inhibitor |
|  | Aminosalicylate sodium (10 μM) | 6.656 | 0.517 | Anti-tubercular agent |
|  | Benzoic acid (1.1 μM) | 6.581 | 0.513 | Anti-fungal agent |
| 33 μM | Benzoic acid (1.1 μM) | 8.753 | 0.637 | Anti-fungal agent |
|  | Carprofen (1.1 μM) | 8.669 | 0.633 | NSAID (COX-2 inhibitor) |
|  | Carprofen (10 μM) | 8.557 | 0.627 | NSAID (COX-2 inhibitor) |
| 11 μM | Aminosalicylate sodium (3.3 μM) | 9.19 | 0.659 | Anti-tubercular agent |
|  | Aminosalicylate sodium (30 μM) | 9.142 | 0.657 | Anti-tubercular agent |
|  | Valaciclovir HCl (1.1 μM) | 9.077 | 0.653 | Viral DNA polymerase inhibitor |
| 3.7 μM | Aminosalicylate sodium (1.1 μM) | 10.34 | 0.711 | Anti-tubercular agent |
|  | Tropicamide (1.1 μM) | 9.602 | 0.679 | Anti-muscarinic agent |
|  | Amlodipine (1.1 μM) | 9.275 | 0.663 | Calcium channel blocker |

The BioMAP profiles of three compounds were compared to the profiles of reference compounds in the BioMAP database, comprising over 3,000 compounds. The three compounds with the top three Z-score are listed.
